# Human papillomavirus vaccine uptake in adolescence and adherence to cervical cancer screening in Switzerland: a national cross-sectional survey

**DOI:** 10.1007/s00038-017-1050-x

**Published:** 2017-11-06

**Authors:** Monica N. Wymann, Anne Spaar Zographos, Ekkehardt Altpeter, Virginie Masserey Spicher, Nicola Low, Mirjam Mäusezahl-Feuz

**Affiliations:** 1 Communicable Disease Division, Swiss Federal Office of Public Health, Schwarzenburgstrasse 157, 3003 Bern, Switzerland; 2 Institute of Social and Preventive Medicine, University of Bern, Finkenhubelweg 11, 3012 Bern, Switzerland

**Keywords:** HPV vaccination, Cervical cancer screening, Determinants, Vaccine hesitancy, Sexual behavior

## Abstract

**Objectives:**

The objectives were to measure uptake of and factors associated with human papillomavirus (HPV) vaccination initiation and to determine whether HPV vaccination reduced the uptake of cervical cancer screening.

**Methods:**

We conducted a cross-sectional survey in a random sample of Swiss women aged 18–49 years in 2014 (*N* = 3588).

**Results:**

Vaccination initiation was 69.3% and full coverage (three doses) 54.1% for 18–20-year olds, respectively, 42.4% and 33.9% for 21–24-year olds. Women with ≥ 10 lifetime sexual partners were less likely to have received any HPV vaccination than women with ≤ 2 partners (18–20 years OR 0.2, 21–24 years OR 0.5). Amongst 1000 unvaccinated women (18–24 years), reasons for not having initiated vaccination were lack of information (22.5%) and fear of vaccine side effects (18.1%). Vaccination status was not associated with adherence to cervical cancer screening recommendations (OR 1.3). 95.4% of all vaccinated participants knew about the continued need for screening.

**Conclusions:**

Our data suggest that HPV vaccination is not associated with reduced uptake of cervical cancer screening. This study provides information that can be used to improve HPV vaccination uptake in Switzerland.

**Electronic supplementary material:**

The online version of this article (doi:10.1007/s00038-017-1050-x) contains supplementary material, which is available to authorized users.

## Introduction

The aim of human papillomavirus (HPV) vaccination programs is to reduce the incidence of cervical cancer and other HPV-associated cancers (Barr and Sings 2008). Several European countries, including the United Kingdom, Portugal and Denmark have achieved targets of 80% for completed HPV vaccination (ECDC 2012; Public Health England 2015; Statens Serum Institute 2013). In Switzerland, HPV vaccination was included in the National Vaccination Plan in 2007 for 11- to 26-year-old women (Bundesamt für Gesundheit and EKIF 2008). The recommended age for primary HPV vaccination in Switzerland is 11–14 years. The majority of cantons provide the vaccination to this age group through school health services. The national target until 2012 was 80% full coverage for 11–14-year olds and 50% for catch-up vaccination in 15–19-year olds. The Swiss National Vaccination Coverage Survey routinely collects HPV vaccination coverage in 16-year olds (Bundesamt für Gesundheit 2008). Since 2008, each of the 26 cantons (states) in Switzerland has been responsible for organizing its own HPV vaccination program. The cost of HPV vaccination is fully covered by health insurance for vaccinations administered within public health vaccination programs (Bundesamt für Gesundheit 2010a, b). Health insurance also covers Papanicolaou (Pap) screening, the other main measure to control cervical cancer. The Swiss Gynecological Society recommends Pap screening from age 21 or from the start of sexual activity every 2 years. In 30- to 70-year-old women, screening should be performed every 3 years assuming that the last three screening test results were normal. Thereafter, screening is only continued in case of continued sexual activity or previous abnormal screening results (Gynécologie Suisse, Kommission Qualitätssicherung 2012).

Concerns have been raised that HPV-vaccinated women might forgo cervical cancer screening, owing to a false sense of security (ECDC 2012), or that women who are not vaccinated against HPV might also be less likely to attend cancer screening. The objectives of this study were: to determine HPV vaccination initiation and full coverage and the factors associated with initiation of vaccination in Switzerland; to determine the reasons for not being vaccinated against HPV; to see if women know about the need for cervical cancer screening after HPV vaccination; and to determine whether HPV vaccination affects adherence to cervical cancer screening recommendations.

## Methods

We designed a nationally representative cross-sectional questionnaire survey. A professional telephone survey company conducted the recruitment and data collection. The questionnaire included questions on participants’ self-reported HPV vaccination status. Women were asked whether they had used their vaccination record to complete this question or not. The interview team did not have access to the hard copies of the vaccination records. Additional questions included reasons for receiving or not receiving HPV vaccination; sexual behavior; cervical cancer screening behavior and outcome; and lifetime occurrence of genital warts. All questions were multiple choice or open questions. Some questions included a category “other” which could be completed as free text. Our questionnaire was available in French, German and Italian either as telephone survey, as self-administered paper or as online questionnaire. The telephone interview took about 11 min, the online version nine minutes to complete. We piloted the sampling procedure and questionnaire with 227 completed interviews between November 18 and December 4, 2013. Invitation letters for the main survey were sent from January 24, 2014 onwards. The main survey took place between February 29 and May 26, 2014.

### Participants and recruitment

Inclusion criteria for participation in the study were: female sex; aged 18–49 years; address within Switzerland recorded in a commercial directory; and ability to answer questions in German, French or Italian. We used a commercial household directory, which covers about 95% of all Swiss private households, to select households with 18- to 49-year-old women. We used stratified random sampling based on two age classes, 18–24 and 25–49 years, three language regions and municipality size. We sent each selected household a letter giving background information. The telephone interview first screened for eligibility by sex and age. For households without a registered telephone number, the information letter asked a woman aged 18–49 years to respond and, if there was more than one eligible woman, we asked the woman who most recently had her birthday to participate (Hüfken 2003). We offered an incentive of CHF 10 for either a self-completed mailed or online response. If the questionnaire was not completed within 2 weeks, a reminder was sent.

We aimed to interview 2250 women who had been eligible for primary or catch-up vaccination after cantonal vaccination programs had started. At the time of the survey in 2014, girls aged 11–14 years in 2008 were aged 17–20 and those aged 15–18 years were aged 21–24. We excluded 17-year olds because they were too young to give consent to participate. To obtain comparable data about cervical cancer screening behavior, we included older women aged 25–49 years; target sample size for this age class was 1125.

### Statistical analyses

We applied three sampling weights to generate estimates that were nationally representative. First, inverse probabilities for selection of households and women within each household, according to language region, age of household members and existence of a telephone number; second, a weight to account for the 2:1 ratio of younger to older women for each language region and annual age classes; third, a constant factor to correct for the actual sample size. To calculate response rate, we divided the number of completed interviews by the estimated number of eligible households, based on guidelines from the Council of American Survey Research Organizations (CASRO 1982).

We present descriptive statistics with 95% confidence intervals (CI); numbers are the unweighted totals and percentages are those after applying the survey weights. Of the 3622 completed interviews we excluded 34 with impossible values for age at vaccination and vaccine type. We did not impute any missing values. We used weighted logistic regression to investigate factors associated with HPV vaccination and cervical cancer screening behaviors. We stratified analyses for vaccination initiation by age class 18–20 years and 21–24 years (and report results for completed vaccination in Supplementary Table 5). In the models about adherence to cervical cancer screening recommendations by the Swiss Gynecological Society, we focused on sexually active women aged 20–49 years only; adherence to guidelines was considered to be present if 20- to 29-year-old women had a Pap test every 2 years or more often; 30- to 49-year-old women every 3 years or more often (Gynécologie Suisse 2012). For both outcomes, vaccination and screening, we examined the same independent categorical variables: language region (German, French, Italian); migratory background (yes, no); education (primary and lower secondary, upper secondary and post-secondary non-tertiary, undergraduate and post-graduate); household income (CHF < 5000, 5000–8999, ≥ 9000); smoking (never, current and stopped), age at first sexual intercourse (< 18 years, ≥ 18 years), number of lifetime sexual partners (0–2, 3–9, ≥ 10) and use of hormonal contraception (no, yes, not yet sexually experienced). In addition, we included initiation of vaccination (yes, no) as an independent variable in the screening model and initiation of cervical cancer screening (yes, no) in the vaccination model. We report results for univariable and multivariable models as odds ratios (OR) with 95% CI. We performed all the analyses with the survey function of Stata/SE 14.0 for Windows (StatCorp LP, College Station, TX, USA).

## Results

We published a German and French language summary of the methods and descriptive results in our surveillance bulletin in 2015 (Bundesamt für Gesundheit 2015a, b).

### Response rate, demographic characteristics and sexual behavior

**Fig. 1:**
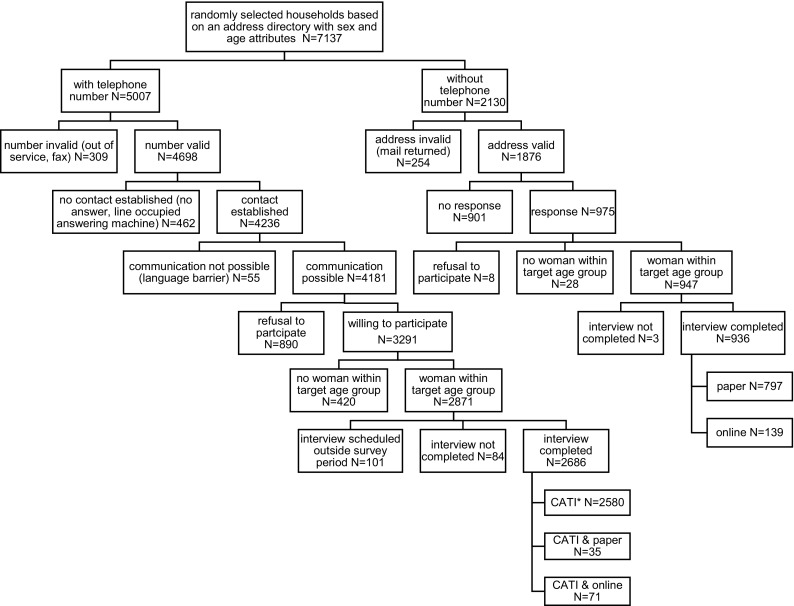
Enrollment process of the participants in the Swiss human papillomavirus survey (Switzerland 2014). Asterisk indicates that *CATI* is an abbreviation for computer assisted telephone interview

We randomly selected 7137 households, 5007 with and 2130 without registered telephone numbers. We obtained 3622 completed interviews, 2446 of these were in the age group 18–24 years and 1176 in the age group 25–49 years (Fig. 1). The estimated overall response rate for eligible women was 66.2%; 76.3% for those with registered telephone numbers and 57.2% for those without telephone numbers. We analyzed 3588 interviews, 2414 with 18–24-year olds and 1174 with 25–49-year olds. We excluded 34 interviews from analysis because vaccination dates were implausible, e.g., vaccination date before date of approval of the HPV vaccines in Switzerland or incorrect brand name of vaccine provided. The median age was 21 years in the 18–24-year olds and 40 years in the 25–49-year olds; median number of lifetime sexual partners was 2 for the former and 4 for the latter age group. We provide further demographic characteristics and sexual behavior reported by the participating women in Supplementary Tables 1 and 2.

### HPV vaccination coverage and reasons for not being vaccinated

**Fig. 2:**
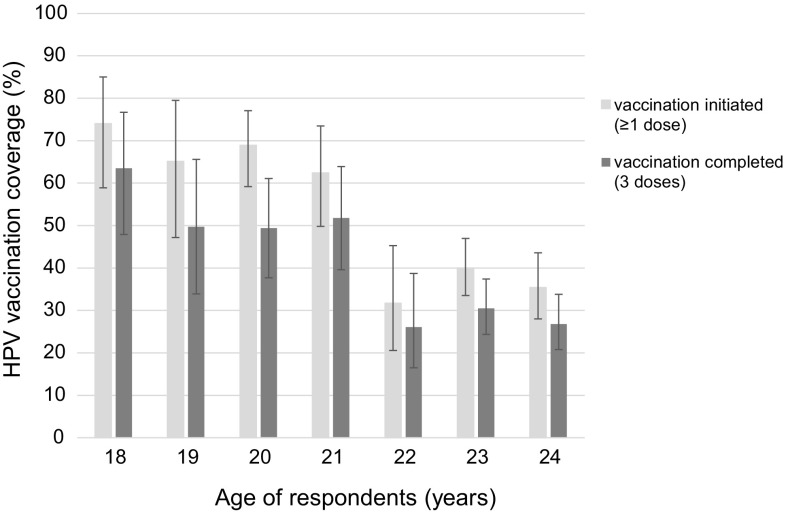
Age-trends in human papillomavirus vaccination initiation and full coverage in 18–24-year olds (*n* = 2363) (Swiss human papillomavirus survey, Switzerland 2014)

Table 1 provides information about HPV vaccination. Amongst 18- to 20-year-old women in 2014 (aged 12–14 years in 2008), 69.3% (95% CI 61.1–76.5%) had initiated HPV vaccination (≥ 1 dose) and 54.1% (95% CI 45.4–62.6%) had received three doses. Of the 21–24-year olds (aged 15–18 years in 2008), 42.4% (95% CI 36.8–48.2%) had initiated and 33.9% (95% CI 28.7–39.4%) had completed HPV vaccination. Vaccination coverage was highest in women aged 18 years in 2014 (74.0%, 95% CI 58.9–85.0% for initiation and 63.5%, 95% CI 47.9–76.7% for completion) (Fig. 2). 97.2% (95% CI 94.7–98.5%) of vaccinations done at the recommended age of 11–14 years were administered before first sexual intercourse. In the age group 25–49 years, 2.5% (95% CI 1.4–4.5%) had initiated and 1.6% (95% CI 0.8–3.3%) completed vaccination. In the group of women aged 18–24 years, 39.3% used their vaccination records to confirm vaccination details and in the group aged 25–49 years, 14.6% checked their vaccination records.

**Table 1:** Human papillomavirus vaccination status reported by women aged 18–24 years (Swiss human papillomavirus survey, Switzerland 2014)

	*N*	Percentage, median	95% CI or SD (%)
Number of HPV vaccine doses	2363		
0 dose		46.6%	41.6–51.6
1 dose		5.4%	3.6–8.0
2 doses		4.7%	3.3–6.6
3 doses		41.3%	36.4–46.3
Vaccinated, number of doses unknown		2.2%	1.6–2.9
HPV vaccination initiated, by language region	2363		
French		68.1%	60.6–74.8
German		47.4%	41.1–53.9
Italian		63.3%	51.6–73.6
Age at first HPV vaccine dose	1243		
11–14 years		39.8%	32.7–47.5
15–19 years		54.4%	47.2–61.4
≥ 20 years		5.8%	4.1–8.1
Median		15 years	
Vaccine type(s) received	826		
Quadrivalent		95.0%	81.7–97.0
Bivalent		3.5%	2.1–5.9
Quadrivalent and bivalent		1.5%	0.4–4.9
Timing of vaccination at age 11–14 years in relation to first sexual intercourse	470		
Before first sexual intercourse		97.2%	94.7–98.5
Same year		2.3%	1.2–4.3
After first sexual intercourse		0.5%	0.1–3.5
Timing of catch-up vaccination at age 15–19 years in relation to first sexual intercourse	716		
Before first sexual intercourse		49.7%	42.4–56.9
Same year		19.1%	14.3–25.1
After first sexual intercourse		31.2%	25.6–37.4

*CI* confidence interval; *SD* standard deviation. All estimates are weighted. The denominator *N* (unweighted) varies across variables because of item non-response and questions restricted to subgroups

In Table 2, we present the reasons provided by the 1000 women aged 18–24 years with zero HPV vaccine doses for not having initiated HPV vaccination (participants could give more than one reason). The two major reasons given by 18–20-year olds (*N* = 321) were fear of vaccine side effects (30.3%, 95% CI 16.8–48.3%) and being opposed to HPV vaccination (20.7%, 95% CI 10.5–36.9%). In the age group 21–24 years (*N* = 679), the two major reasons were lack of information (24.7%, 95% CI 18.3–32.3%) and having had already sexual partners (21.2%, 95% CI 14.4–30.0%).

**Table 2:** Reasons given by Swiss women aged 18–24 years for not having initiated human papillomavirus vaccination (multiple answers permitted) (Swiss human papillomavirus survey, Switzerland 2014)

	18–20 years^a^		21–24 years^b^		18–24 years (combined)	
	%	95% CI	%	95% CI	%	95% CI
Not (sufficiently) informed about HPV vaccination	16.9	8.9–29.7	24.7	18.3–32.3	22.5	17.2–28.9
Fear of vaccine side effects	30.3	16.8–48.3	13.5	8.8–20.2	18.1	12.5–25.5
Had already one or more sexual partners	3.6	1.7–7.4	21.2	14.4–30.0	16.4	11.3–23.2
Opposed to vaccination in general	20.7	10.5–36.9	11.5	7.0–18.3	14.0	9.3–20.5
HPV vaccination is for younger girls/too old^c^	0.6	0.2–2.1	15.9	9.7–25.1	11.7	7.1–18.8
No time or forgotten	11.6	7.2–18.1	10.1	7.4–13.6	10.5	8.1–13.5
Physician advised against vaccination	5.4	2.9–9.8	8.0	5.5–11.6	7.3	5.3–10.0
Friends/family advised against vaccination	12.0	6.1–22.3	3.4	2.2–5.3	5.8	3.7–8.8
Thinks vaccine does not work	4.9	2.7–8.6	3.1	1.9–4.9	3.6	2.5–5.1
Would rather rely on cervical cancer screening than vaccination	2.1	7.9–5.5	3.4	2.1–5.5	3.1	2.0–4.7
Other reasons	14.9	9.8–22.2	13.8	10.5–18.0	14.1	11.2–17.6
Refusal or do not know	4.0	2.2–7.1	3.7	2.3–5.8	3.8	2.6–5.4

All estimates are weighted. *CI* confidence interval

^a^Target group for primary vaccination in 2008 (aged 12–14 years), *N* = 321

^b^Target group for catch-up vaccination in 2008 (aged 15–18 years), *N* = 679

^c^Based on their year of birth, all women had been within the target age range for HPV vaccination in 2008

### Factors associated with HPV vaccination

Table 3 shows factors associated with initiation of HPV vaccination. Women who reported ≥ 10 lifetime partners in 2014 were less often vaccinated than women who reported 0–2 partners (18–20 years OR 0.2, 95% CI 0.0–0.5; 21–24 years OR 0.5, 95% CI 0.3–1.0). Women in the French-speaking region were more likely to initiate vaccination than those in the German-speaking region (18–20 years OR 2.5, 95% CI 1.3–4.8; 21–24 years OR 3.1, 95% CI 2.1–4.6). In 21–24-year olds, initiation of catch-up vaccination was also more frequent in the Italian-speaking than in the German-speaking region (OR 2.5, 95% CI 1.1–5.7). In this age group, women with the highest level of household income had an OR of 2.4 (95% CI 1.5–3.8) for having started catch-up vaccination compared with women with the lowest income. Women aged 21–24 years using hormonal contraception were more likely to have initiated vaccination than those not using hormonal contraception (OR 1.5, 95% CI 1.0–2.3). In both age groups, we observed no association between cervical cancer screening and initiation of vaccination (18–20 years, OR 1.3, 95% CI 0.6–2.7; 21–24 years OR 1.1, 95% CI 0.6–1.9). Results of logistic regression analysis for factors associated with completed vaccination are given in Supplementary Table 5.

**Table 3:** Initiation of human papillomavirus vaccination in 18- to 24-year-old women: results of univariate and multivariate analysis (Swiss human papillomavirus survey, Switzerland 2014)

	18–20 years						21–24 years					
	*N*_yes_/*N*_obs_^a^	Weighted proportion %	Univariable model		Multivariable model		*N*_yes_/*N*_obs_	Weighted proportion %	Univariable model		Multivariable model	
			OR	95% CI	OR	95% CI			OR	95% CI	OR	95% CI
Language region												
German	314/404	64.2	1	(Reference)	1	(Reference)	303/531	36.2	1	(Reference)	1	(Reference)
French	264/417	81.8	2.5	1.2–5.3	2.5	1.3–4.8	204/540	58.0	2.4	1.5–4.0	3.1	2.1–4.6
Italian	136/216	73.7	1.6	0.7–3.5	1.6	0.7–3.8	125/256	55.5	2.2	1.1–4.5	2.5	1.1–5.7
Migratory background												
No	649/938	69.6	1	(Reference)	1	(Reference)	548/1136	44.1	1	(Reference)	1	(Reference)
Yes	64/97	66.4	0.9	0.3–2.4	0.4	0.2–1.2	84/191	32.5	0.6	0.3–1.2	0.7	0.4–1.2
Education												
Primary and lower secondary	345/498	71.0	1	(Reference)	1	(Reference)	45/102	25.3	1	(Reference)	1	(Reference)
Upper secondary and post-secondary non-tertiary	164/238	73.3	1.1	0.6–2.2	0.9	0.4–1.8	203/518	38.1	1.8	0.5–6.5	1.3	0.5–3.0
Undergraduate and post-graduate	202/296	63.2	0.7	0.3–1.8	0.5	0.2–1.1	383/701	49.1	2.8	0.8–10.1	2.0	0.8–4.8
Household income (CHF)												
< 5000	211/308	76.0	1	(Reference)	1	(Reference)	206/503	31.3	1	(Reference)	1	(Reference)
5000–8999	132/186	69.0	0.7	0.2–2.0	1.1	0.4–2.8	112/228	44.0	1.7	0.9–3.2	1.6	0.9–2.6
≥ 9000	184/256	67.7	0.7	0.3–1.6	1.0	0.5–2.1	185/343	52.1	2.4	1.3–4.3	2.4	1.5–3.8
Unknown	157/243	63.4	0.5	0.2–1.3	0.9	0.5–1.8	94/178	41.1	1.5	0.8–3.1	1.7	0.9–3.0
Smoking												
Yes or stopped	210/302	75.3	1	(Reference)	1	(Reference)	217/542	33.6	1	(Reference)	1	(Reference)
Never	503/733	67.5	0.7	0.4–1.3	0.8	0.4–1.5	414/782	49.8	2.0	1.2–3.2	1.6	1.0–2.5
Age at first sexual intercourse												
< 18 years	432/596	72.4	1	(Reference)	1	(Reference)	408/851	40.5	1	(Reference)	1	(Reference)
≥ 18 years	282/440	65.5	0.7	0.3–1.5	0.7	0.3–1.5	224/476	46.0	1.3	0.8–1.9	0.8	0.5–1.3
Number of lifetime sexual partners												
0–2	302/440	72.6	1	(Reference)	1	(Reference)	219/425	47.2	1	(Reference)	1	(Reference)
3–9	201/290	65.1	0.7	0.3–1.6	0.5	0.3–1.0	268/585	43.7	0.9	0.6–1.4	1.0	0.7–1.6
≥ 10	211/306	46.5	0.3	0.1–1.4	0.2	0.0–0.6	145/317	27.2	0.4	0.2–0.9	0.5	0.3–1.0
Cervical cancer screening												
Yes	319/480	61.8	1	(Reference)	1	(Reference)	93/205	43.6	1	(Reference)	1	(Reference)
No	382/537	76.1	2.0	1.0–4.0	1.3	0.6–2.7	522/1078	42.4	1.0	0.6–1.6	1.1	0.6–1.9
Use of hormonal contraception												
No	116/175	62.5	1	(Reference)	1	(Reference)	129/309	34.2	1	(Reference)	1	(Reference)
Yes	405/572	77.3	2.0	0.9–4.6	1.5	0.7–3.2	421/835	45.1	1.6	1.0–2.4	1.5	1.0–2.3
Not yet sexually experienced	175/259	64.2	1.1	0.3–3.3	6.5	1.1–39.1	53/114	48.4	1.8	1.0–3.4	2.8	1.0–7.8

*OR* odds ratio, *CI* confidence interval

^a^The denominator *N*_obs_ varies across variables due to item non-response

### Factors associated with cervical cancer screening, screening behavior and knowledge about screening recommendations after HPV vaccination

When asked about the necessity for cervical cancer screening after HPV vaccination, 75.2% (95% CI 67.6–81.4%) of all vaccinated women (*N* = 1385) stated that cervical cancer screening should be continued at the same frequency; 20.3% (95% CI 14.2–28.1%) thought that screening is still needed after HPV vaccination but less often; 0.8% (95% CI 0.5–1.4%) considered screening unnecessary after HPV vaccination; and 3.8% (95% CI 2.6–5.5%) did not know. Among unvaccinated women (*N* = 2111), the values were 56.2% (95% CI 52.1–60.0%), 30.2% (95% CI 27.3–34.5%), 1.5% (95% CI 0.8–2.9%) and 12.0% (95% CI 9.4–14.4%), respectively.

In the age group 18–24 years (*N* = 2345), 71.7% had had at least one Pap test; the value was 72.7% (95% CI 65.2–79.1) for women not having initiated HPV vaccination and 69.9% (95% CI 62.7–76.2%) for vaccinated women. 98.1% of women aged 25–49 years (*N* = 1141) had had at least one Pap test (Supplementary Table 3). Amongst women having initiated Pap testing, 77.7% (95% CI 73.2–81.7%) of 18–24-year olds and 65.0% (95% CI 60.8–68.9%) of 25–49-year olds had Pap tests annually, which is more often than recommended by the expert guidelines.

In multivariable analysis, which focused on sexually active women aged 20–49 years, we found that having been vaccinated against HPV was not associated with adherence to cervical cancer screening recommendations (OR 1.3, 95% CI 0.8–2.1). Besides age, household income and use of hormonal contraception affected adherence to cervical cancer screening recommendations. Women with high income had an OR of 2.3 (95% CI 1.2–4.6) to adhere to screening recommendations compared with women with low income. Women using hormonal contraception were more likely to adhere to screening recommendations than those not using hormonal contraception (OR 3.5, 95% CI 1.8–6.6). We provide complete results of multivariable analysis of cervical cancer screening and additional information on mean age at first Pap test, timing of first Pap test in relation to first sexual intercourse, results of cervical cancer screening and frequency of operations at the cervix in Supplementary Tables 3 and 4. Of the 18–24-year olds (*N* = 725) who had never had a Pap test, only 0.5% (95% CI 0.1–2.1%) reported being vaccinated against HPV as a reason for not having initiated Pap testing.

## Discussion

Our survey shows that reported levels of completed HPV vaccination amongst women aged 18–24 years in 2014 were below the target of 80%. Regional differences in initiation of vaccination were present and women who reported higher numbers of sexual partners when they were interviewed in 2014 were less likely to have initiated HPV vaccination. The main reasons that women gave for not being vaccinated were fear of vaccine side effects and lack of information. HPV vaccination initiation was not associated with a reduction in the subsequent uptake of cervical cancer screening.

### Strengths and limitations

Major strengths of this study are its national coverage and the wide range of topics concerning HPV vaccination, including factors associated with vaccination and reasons for lack of vaccination, obtained from the same study population. In the absence of a vaccine registry, surveys offer a way to investigate HPV vaccine uptake and cervical cancer screening in detail. The survey also has several limitations. First, the data are self-reported and vaccination status was confirmed by written records for only a minority of women. Reported vaccination coverage was, however, in line with the findings of routine Swiss vaccination coverage surveys (SNVCS), which record national coverage by age 16 years of 51% for completion (three doses) during the survey period 2011–2013 (Bundesamt für Gesundheit 2015a, b). Second, the sampling frame did not reach all eligible women with the same probability. The response of 76% was adequate for women interviewed by telephone but lower for women without registered telephone number (57%). We found that women aged 18–24 years with a registered telephone number who completed at least part of the interview by CATI reported higher vaccination coverage than those without registered telephone number doing the interview either online or on paper. But based on our design, we cannot say if this observed difference is due to the interview mode or to other factors associated with having a registered telephone number. To attenuate some of the potential biases introduced by the sampling procedure and study design, we used a complex weighting procedure. Third, questions about socioeconomic factors were asked up to 8 years after vaccination. This delay could bias results if socioeconomic position had changed for a large proportion of women. Finally, women of migrant background were underrepresented.

### Implications for vaccination programs

HPV vaccination uptake in Switzerland, 6 years after the programs started in 2008, did not reach the targets that had been set for either primary or catch-up vaccination. Many other European countries (ECDC 2012) and the USA (Williams et al. 2016) have also had lower coverage than expected. Our survey also shows the lower uptake of HPV vaccination by women in the German than French or Italian language regions in Switzerland. An evaluation of HPV vaccination programs in Switzerland showed higher vaccination coverage levels in cantons in which school health services are involved in the delivery of vaccination for the main age group of 11- to 14-year-old children. In cantons in which school-based delivery is available, coverage was around 60%, compared with around 40% in cantons without during the survey period 2011–2013 of the SNVCS (Spaar and Masserey 2015); such programs are more common in French than in German-speaking cantons (Bundesamt für Gesundheit 2010a, b; Jeannot et al. 2012). An international systematic review of HPV vaccine uptake in adolescents confirms the higher coverage achieved by school-based vaccination programs (Kessels et al. 2012). A mathematical modeling study from the USA, where heterogeneity in vaccination coverage between states (20–57%) is similar to that in Switzerland suggests that expansion of coverage would have the greatest health impact in states with the lowest coverage (Durham et al. 2016). Encouraging school health service involvement in all cantons, but particularly in those with the lowest vaccination coverage, could improve vaccination coverage nationally.

A recent meta-analysis did not find strong evidence for differences in HPV vaccination initiation by parental education or income (Fisher et al. 2013). In our survey, we found no influence of socioeconomic factors in the target group for vaccinations at 11–14 years. However, in the target group for catch-up vaccinations, women with lower income were less likely to be either vaccinated or screened, constituting a risk group for development of cervical cancer.

Understanding reasons for not being vaccinated at the individual level can indicate ways in which vaccination coverage could be improved and disparities reduced. Two of the most commonly cited reasons for not being vaccinated were lack of information and fear of vaccine side effects. These are factors that could be dealt with by improvements in public and school-based information campaigns. One in five 18–20-year olds said that they were not vaccinated against HPV because of opposition to vaccination in general or HPV vaccination in particular, presumably reflecting their parents’ decision. Dialog-based interventions might help to overcome part of this vaccine hesitancy (Jarrett et al. 2015).

### HPV vaccination and sexual behavior

Our survey shows the importance of maximizing HPV vaccination uptake before sexual debut because there are no good predictors of future risky sexual behavior. Women in our survey who had the highest numbers of lifetime sexual partners (≥ 10) at the time they were interviewed were those who were least likely to have received HPV vaccination when they were younger (47%). A survey in Australia found a similar association; women aged 20–29 years with 10 or more lifetime partners were less likely than those with fewer than two partners to have received any HPV vaccine (OR 0.54, 0.31–0.96) (Canfell et al. 2015). In Australia, however, 75% of women with the highest number of sexual partners had initiated vaccination. The observed association between HPV vaccination and lower numbers of future sexual partners should further allay fears that HPV vaccination increases unsafe sexual behavior and confirms that HPV vaccination in girls does not lead to increases in adverse outcomes of sexual activity (Bednarczyk et al. 2012).

### Role of gynecologists in HPV vaccination

For women beyond school age in Switzerland, gynecologists are the main contact persons for the provision of HPV vaccination: Many young women go to a gynecologist for prescriptions for hormonal contraception, which provides opportunities for catch-up HPV vaccination (Canfell et al. 2015). Overall, gynecologists had delivered a quarter of the catch-up vaccinations in Switzerland (Bundesamt für Gesundheit 2015a, b). They are well placed to publicize the recommendation for HPV vaccination irrespective of sexual experience for women below 20 years and to fill the information gaps and alleviate doubts about vaccine safety. Visits to a gynecologist were associated with an increased uptake of HPV vaccination in a population-based survey in Germany (Poethko-Müller et al. 2014).

### HPV vaccination and cervical cancer screening

HPV vaccination was not associated with reduced adherence to cervical cancer screening recommendations amongst Swiss women. The women in our survey also had high levels of knowledge about the continued need for cervical cancer screening after HPV vaccination, confirming findings amongst US women (Anhang Price et al. 2011). Our survey found that cervical cancer screening practices could be improved in Switzerland. A majority of women reported that they had Pap testing more often than recommended, i.e., annually instead of every 2 or 3 years, half had their first Pap test before the recommended age of 20 years; and nearly one-third had a Pap test before their first sexual intercourse, which is clearly against recommendations and does not reduce cervical cancer cases (Moscicki 2010). New technologies for HPV vaccination and cervical cancer screening, including two-dose vaccination schedules, the introduction of a nonavalent HPV vaccine and HPV testing of cytology specimens should be coordinated to maximize cervical cancer prevention efforts for all women.

### Conclusions

Cross-sectional studies that provide detailed data about HPV vaccination, sexual behavior and cervical cancer screening behavior in nationally representative samples of the population are important sources of information for improving vaccination programs and for modeling studies of the predicted impact of HPV vaccination. This study demonstrates a large potential for improving HPV vaccine uptake in Switzerland. It will be important to tackle regional disparities in HPV vaccination coverage as well as information gaps and doubts about vaccine safety. Our data suggest that HPV vaccination is not associated with a reduced uptake of cervical cancer screening. This study provides information that can be used to improve HPV vaccination uptake in Switzerland.

## Electronic supplementary material

Below is the link to the electronic supplementary material.

Supplementary material 1 (DOCX 51 kb)
