# Supplementary material for: Human papillomavirus vaccine uptake in adolescence and adherence to cervical cancer screening in Switzerland: a national cross-sectional survey
Source: Int J Public Health. 2017 Nov 6;63(1):105–14. doi: 10.1007/s00038-017-1050-x (PMC5766720; doi:10.1007/s00038-017-1050-x)
Supplement: Supplementary file 1 — Supplementary material 1 (DOCX 51 kb) [file 38_2017_1050_MOESM1_ESM.docx]

**International Journal of Public Health**

**Supplemental Material: Human papillomavirus vaccine uptake in adolescence and adherence to cervical cancer screening in Switzerland: a national cross-sectional survey**

Monica N. Wymann^1^, Anne Spaar Zographos^1^, Ekkehardt Altpeter ^1^, Virginie Masserey Spicher^1^, Nicola Low^2^, Mirjam Mäusezahl-Feuz^2^

1 Communicable Disease Division, Swiss Federal Office of Public Health, Bern, Switzerland.

2 Institute of Social and Preventive Medicine, University of Bern, Bern, Switzerland.

**Address correspondence to:**

Monica N. Wymann

Email: [monica.wymann@bag.admin.ch](mailto:monica.wymann@bag.admin.ch)

This supplemental document contains the demographic distribution of the study population (Table S1), data on sexual and contraceptive behavior reported by the participating women (Table S2), data on cervical cancer screening behavior and cervical anomalies reported by the participating women (Table S3) and the results of univariate and multivariate analysis for the determinants of compliance with cervical cancer screening recommendations (Table S4) and the determinants of completed HPV vaccination (3 doses) (Table S5).
